# Irisin Serum Levels and Skeletal Muscle Assessment in a Cohort of Charcot-Marie-Tooth Patients

**DOI:** 10.3389/fendo.2022.886243

**Published:** 2022-05-12

**Authors:** Graziana Colaianni, Angela Oranger, Manuela Dicarlo, Roberto Lovero, Giuseppina Storlino, Patrizia Pignataro, Antonietta Fontana, Francesca Di Serio, Angelica Ingravallo, Giuseppe Caputo, Alfredo Di Leo, Michele Barone, Maria Grano

**Affiliations:** ^1^ Department of Emergency and Organ Transplantation, University of Bari, Bari, Italy; ^2^ Department of Basic Medical Sciences, Neuroscience and Sense Organs, University of Bari, Bari, Italy; ^3^ Clinical Pathology Unit, Polyclinic of Bari, Bari, Italy; ^4^ Gastroenterology Unit, Department of Emergency and Organ Transplantation, University of Bari, Bari, Italy; ^5^ Territorial Neurology Service of Parkinson Disease and Movement Disorders Network - Apulia - Azienda Sanitaria Locale (ASL) Bari, Bari, Italy

**Keywords:** myokine, irisin, osteoporosis, muscle atrophy, CMT

## Abstract

**Background:**

Charcot-Marie-Tooth (CMT) indicates a group of inherited polyneuropathies whose clinical phenotypes primarily include progressive distal weakness and muscle atrophy. Compelling evidence showed that the exercise-mimetic myokine irisin protects against muscle wasting in an autocrine manner, thus possibly preventing the onset of musculoskeletal atrophy. Therefore, we sought to determine if irisin serum levels correlate with biochemical and muscle parameters in a cohort of CMT patients.

**Methods:**

This cohort study included individuals (N=20) diagnosed with CMT disease. Irisin and biochemical markers were quantified in sera. Skeletal muscle mass (SMM) was evaluated by bioelectric impedance analysis, muscle strength by handgrip, and muscle quality was derived from muscle strength and muscle mass ratio.

**Results:**

CMT patients (m/f, 12/8) had lower irisin levels than age and sex matched healthy subjects (N=20) (6.51 ± 2.26 vs 9.34 ± 3.23 μg/ml; p=0.003). SMM in CMT patients was always lower compared to SMM reference values reported in healthy Caucasian population matched for age and sex. Almost the totality of CMT patients (19/20) showed low muscle quality and therefore patients were evaluated on the basis of muscle strength. Irisin was lower in presence of pathological compared to normal muscle strength (5.56 ± 1.26 vs 7.67 ± 2.72 μg/ml; p=0.03), and directly correlated with the marker of bone formation P1PN (r= 0.669; 95%CI 0.295 to 0.865; p=0.002), but inversely correlated with Vitamin D (r=-0.526; 95%CI -0,791 to -0,095; p=0.017). Surprisingly, in women, irisin levels were higher than in men (7.31 ± 2.53 vs 5.31 ± 1.02 μg/ml, p=0.05), and correlated with both muscle strength (r=0.759; 95%CI 0.329 to 0.929; p=0.004) and muscle quality (r=0.797; 95%CI 0.337 to 0.950; p=0.006).

**Conclusion:**

Our data demonstrate lower irisin levels in CMT patients compared to healthy subjects. Moreover, among patients, we observed, significantly higher irisin levels in women than in men, despite the higher SMM in the latter. Future studies are necessary to establish whether, in this clinical contest, irisin could represent a marker of the loss of muscle mass and strength and/or bone loss.

## Introduction

Charcot-Marie-Tooth disease (CMT) is a group of hereditary genetic disorders characterized by progressive neuropathy affecting both the motor and sensory nerves (1, 2). Although CMT includes more than 100 disease-associated genes with a number of different mutations in each that affect nerve structure and function, a basic classification categorizes all CMT neuropathies into two main subtypes: demyelinating neuropathy type 1 (CMT1) and axonal neuropathy type 2 (CMT2) (3). Clinical phenotypes primarily include slowly progressive distal weakness and muscle atrophy, hypo/areflexia, and skeletal deformities. Currently, there are no effective treatment approaches other than treating symptoms with medications such as nonsteroidal anti-inflammatory drugs that provide relief of lower back or leg pain (4). Rehabilitative management of patients with CMT has shown that physical activity can provide a moderate increase in muscle strength and musculoskeletal function (5). However, the muscle deficits that characterize these patients limit their daily activities, leading to severe disability, such that a program of moderate physical activity cannot always be considered as a rehabilitative treatment for CMT. In fact, clinical manifestations of these patients include cramping in the calves, forefoot stumbling, ankle twisting, poor knee control, and frequent falls to the need for a wheelchair (5). Benefits of exercise on the musculoskeletal system are widely recognized as primary non-pharmacologic intervention for several diseases (6). However, recent findings show that the positive outcome of physical activity is achieved not only by the mechanical load applied on the musculoskeletal system, but also *via* biochemical signals, the myokines, secreted during contraction that act locally or systemically on several body districts. Therefore, the revolutionary discovery of these myokines would expand the search for exercise-mimetic drugs that could be widely used to treat patients who suffer from immobilization and cannot perform physical activity. Among these, irisin is a hormone-peptide synthesized from skeletal muscle performing key actions on the whole-body metabolism (7–9). Our previous study showed that intermittent administration of irisin in healthy young mice increases cortical bone mass and makes the skeleton more resistant to fractures (10). In humans, we have documented with several studies an existing positive association between circulating levels of irisin and bone mineral density (11–15). Compelling evidence exists for a link between irisin and skeletal muscle mass. In a mouse model of disuse-induced muscular atrophy, we showed that irisin treatment prevents muscle wasting and mitochondrial dysfunction during musculoskeletal unloading (16). Another study found that irisin acts as pro-myogenic factor by inducing skeletal muscle hypertrophy and rescuing denervation-induced atrophy (17). At our knowledge, in the literature there are few studies in humans and some data have shown that irisin levels may be predictive of sarcopenia (18–20). Given the observed low correlation between muscle strength and serum irisin levels, it is relevant that further investigation be pursued to confirm the data achieved in mouse models.

Few studies have investigated the possible key determinants involved in muscle mass and function in CMT patients. In this study, we evaluated irisin serum levels in a cohort of CMT patients and its possible association with other biochemical parameters influencing muscle mass and function.

Our hypothesis was based on compelling evidence demonstrating that the myokine irisin prevents the onset of musculoskeletal decline, a clinical manifestation suffered by Charcot-Marie-Tooth patients. Therefore, possible application for the study findings could be the use of irisin as a biomarker of the loss of muscle mass and strength and possibly a new therapeutic approach to improve musculoskeletal function in patients with CMT.

## Material and Methods

### Standard Protocol Approvals, Registrations, and Patient Consents

Twenty CMT patients were selected by the territorial neurology service at the department of Physical Medicine and Rehabilitation (ASL, Bari, Italy) in collaboration with the Charcot Marie Tooth Italian Association (AICMT) and underwent an evaluation of their muscle mass and strength as outpatient at the Clinical Nutrition section of the Gastroenterology Unit, Department of Emergency and Organ Transplantation, Policlinic University Hospital, Bari (Italy) from November 2019 to October 2020. The study was conducted in compliance with the Declaration of Helsinki and the International Conference on Harmonization Principles of Good Clinical Practice. All participants gave informed consent allowing their anonymized information to be used for a data analysis. The research protocol was approved by the local Ethics Committee (Project Iri-mCMT N. 6012) and registered on www.ClinicalTrial.com (NCT04786522). The main exclusion criteria were taking anti-osteoporosis therapy, and, because renal dysfunction is also known to affect irisin levels, subjects were also excluded if they had renal failure with glomerular filtration rate <30 mL/min. Regarding comorbidities, 2 patients were affected by type II diabetes, 3 patients were affected by Hashimoto’s thyroiditis of which only one was receiving pharmacological treatment, and 1 patient was using wheelchair. All patients were used to engaging in moderate physical activity in the capacity allowed by the disease. No study subjects changed their lifestyle (physical activity and diet) at least 3 months before enrollment. At enrollment, we collected the following data for each patient: sex, age, weight and height. Muscle strength and body composition were evaluated by handgrip dynamometer and bioelectrical impedance analysis, respectively. Serum irisin levels of controls were obtained from a historical control group of healthy subjects matched for age, sex and BMI, obtained in our ongoing population-based study (unpublished data).

### Evaluation of Muscle Strength

The handgrip dynamometer (Jamar, Sammons Preston, Bolingbrook, IL, USA) was used to determine the isometric force of the forearm. The test procedure was carried out as previously described (21). Patients repeated the evaluation three consecutive times for each forearm, and the mean value obtained from the three tests was used (22). As described in detail by Mathiowetz (23), patients were invited to use the dynamometer with their shoulder adducted and neutrally rotated, elbow flexed at 90 degrees, forearm in neutral position, and wrist between 0- and 30-degrees dorsiflexion using their dominant hand. Three successive trials were recorded for each hand, with an interval of 15 seconds between each trial. The coefficient of variation between trials was lower than ± 5%. The cut-offs considered to diagnose the reduction in muscle strength were <27 Kg in men and <16 Kg in women, as previously reported (24).

### Evaluation of Body Composition

The measurement of body composition was performed using Bioimpedance (BIA 101, Akern srl, Pontassieve (FI), Italy) as previously described (21). Briefly, all measurements were performed between 9:00 and 10:00 a.m. after an overnight fast and in supine position with arms and legs abducted from the body. Touch-type electrodes were attached on the dorsum of the dominant hand and foot, one near the phalanges and the other more proximally, 5 cm away from the other. All evaluations were performed after 5 minutes of rest in a room with a temperature set between 24 and 26°C. The data on resistance and reactance were interpreted by special software (Pro Bodygram 3.0, Akern srl), which provided the fat mass (FM), fat-free mass (FFM), and skeletal muscle mass (SMM) expressed as kg, as well as muscle quality [muscle strength (kg)/(skeletal muscle mass (kg)] of each patient. SMM was compared with the normal value reported in literature in subject matched for sex and age (25).

### Biochemical Measurements

Fasting blood samples were obtained in the morning from 8:30 to 9:30 AM. Serum samples were assayed for calcium, phosphorus, lactate dehydrogenase (Ldh), creatine phosphokinase (Cpk), creatinine, myoglobin, thyroid stimulating hormone (TSH), 25(OH)-Vitamin D, osteocalcin, bone alkaline phosphatase (b-ALP), Procollagen type I N-terminal propeptide (P1NP), C-terminal telopeptide of type I collagen (CTX-I), osteoprotegerin (OPG), Receptor activator of nuclear factor kappa-B ligand (RANK-L), haptoglobin, sclerostin, myostatin, and irisin. Irisin serum concentrations were detected using a competitive ELISA kit (AdipoGen, Liestal, Switzerland) with intra-assay coefficient of variation ≤ 6.9%. The lowest level of Irisin that can be detected is 1ng/ml and the assay range is 0.001-5 µg/mL. Colorimetric reactions were measured by using spectrophotometer (Eon, BioTek, Winooski, Vermont, USA) at the end of each assay.

### Statistical Analysis

For continuous variables, an analysis of sample distribution was performed by evaluating symmetry with the Skewness and Kurtosis tests, and the variables were expressed as mean ± standard deviation (SD) or median and interquartile range (IQR), while dichotomous variables were expressed as a proportion with a 95% confidence interval (95%CI). Unpaired two tailed t-test for mean values and Pearson’s correlation coefficient for linear regression analysis were used to compare parameters with normal distribution. Parameters with non-normal distribution were instead evaluated with the Mann-Whitney test and, for linear regression analysis with the Spearman’s coefficient. Multivariate linear regression was used to investigate the determinants of muscle quality and P1PN. The results were considered statistically significant for p values ≤0.05. Data are presented as box-and-whisker plots with median and interquartile ranges, from max to min, with all data points shown. For linear regression and multivariate regression model, r or β and p values are included into the graph. IBM SPSS statistics version 22 and GraphPad Prism 7.0 were used to analyze data. For correlation analysis, r value < 0.45 was considered low, moderate if r ranges from 0.45 to 0.7, and high if > 0.70.

## Results

### General Characteristics of the Study Population

**Table 1** shows the demographic, anthropometric, skeletal muscle mass, and laboratory parameters of participants. Of these 20 patients, aged 54 ± 14.54 years, 12 (60%) were female. The values of bALP, TSH, Ldh, Cpk, Ca, P, myoglobin, CTX, and P1PN were in the normal range, whereas haptoglobin was higher and osteocalcin was lower than the relative normal ranges in all patients. Creatinine levels were lower than normal ranges in 10 patients, whereas myostatin levels were higher than normal range in 5 patients and lower in 1 patient (**Table S1**).

**Table 1:** Demographic, anthropometric, skeletal muscle mass, and laboratory parameters of patients.

	All patients (N = 20)
**Age** (years)	54 ± 14.54
**Weight** (Kg)	77.84 ± 16.12
**Height** (cm)	163.60 ± 7.10
**BMI** (kg/m^2^)	28.45 (26.05 – 30.25)
**SMM** (Kg)	18.85 (15.85 – 29.25)
**Muscle strength** (Kg)	21.83 ± 11.66
**Muscle quality**	0.99 ± 0.36
**bALP** (μg/L)	17.70 ± 5.64
**TSH** (μIU/ml)	1.35 ± 0.58
**Ldh** (U/L)	158.70 ± 28.28
**Cpk** (U/L)	143.90 ± 71.97
**Ca** (mg/dl)	8.98 ± 0.36
**P** (mg/dl)	3.40 ± 0.49
**Creatinine** (mg/dL)	0.73 ± 0.20
**Myoglobin** (ng/ml)	78.90 ± 32.35
**Haptoglobin** (mg/ml)	7.85 (6.86 – 8.49)
**25(OH)-Vit D** (ng/ml)	20.05 (9.80 – 27,30)
**CTX** (ng/ml)	0.25 ± 0.10
**P1PN** (ng/ml)	43.27 (37.07 – 63.08)
**OPG** (pmol/l)	5.16 ± 1.88
**RANK-L** (ng/ml)	10.80 (5.09 – 28.40)
**Osteocalcin** (ng/ml)	19.03 ± 6.08
**Myostatin** (ng/ml)	4.97 (2.66 – 7.95)
**Sclerostin** (pmol/L)	70.18 ± 26.44

Mean ( ± SD) or Median (25%; 75%) for all variables.

Based on patients with an established genetic diagnosis (75%; N= 15), no significant difference in irisin levels was observed between CMT type1 (N=12) and CMT type2 (N=3) subtypes (6.37 ± 2.07 vs 8.80 ± 3.11 μg/ml; p=0.12).

Finally, 19 of the 20 CMT patients (95%) had 25(OH)-Vitamin D levels under the normal range and were sub-characterized as CMT patients with hypovitaminosis D, including two subjects receiving vitamin D supplementation, which failed to revert deficiency.

In all subjects, irisin serum levels correlated negatively with 25(OH)-Vitamin D (r=-0.526; 95% CI -0,791 to -0,095; p=0.017) and positively with TSH (r=0.470; 95% CI 0.021 to 0.762; p=0.036), P1PN (r=0.645; 95% CI 0.295 to 0.865; p=0.002) and Phosphorous (P) (r=0.583; 95% CI 0.175 to 0.819; p=0.007) levels. No correlation was observed between irisin and the other parameters (**Table 2**).

**Table 2:** Linear Correlation between Irisin and all other parameters (*Spearman Rank Order*).

	Irisin (μg/ml)	
	*r (correlation coefficient)*	*p value*
**Sex**	0.418	*0.065*
**Age** (years)	-0.223	*0.340*
**Weight** (Kg)	0.171	*0.475*
**Height** (cm)	-0.302	*0.205*
**BMI** (kg/m^2^)	0.118	*0.626*
**SMM** (Kg)	-0.358	*0.141*
**Muscle Strength** (Kg)	0.147	*0.541*
**Muscle Quality**	0.420	*0.091*
**bALP** (μg/L)	0.032	*0.891*
**TSH** (μIU/ml)	**0.470**	***0.036***
**Ldh** (U/L)	0.073	*0.758*
**Cpk** (U/L)	0.060	*0.797*
**Ca** (mg/dl)	0.266	*0.252*
**P** (mg/dl)	**0.583**	***0.007***
**Creatinine** (mg/dL)	0.143	*0.542*
**Myoglobin** (ng/ml)	0.065	*0.782*
**Haptoglobin** (mg/ml)	0.221	*0.343*
**25(OH)-Vit D** (ng/ml)	**-0.526**	***0.017***
**CTX** (ng/ml)	0.062	*0.792*
**P1PN** (ng/ml)	**0.669**	***0.002***
**OPG** (pmol/l)	-0.391	*0.087*
**RANK-L** pg/ml	-0.435	*0.055*
**Osteocalcin** (ng/ml)	0.015	*0.947*
**Myostatin** (ng/ml)	-0.017	*0.942*
**Sclerostin** (pmol/L)	-0.097	*0.681*

The bold values are statistically significant.

### Irisin Levels and Muscle Quality

**Figure 1:**
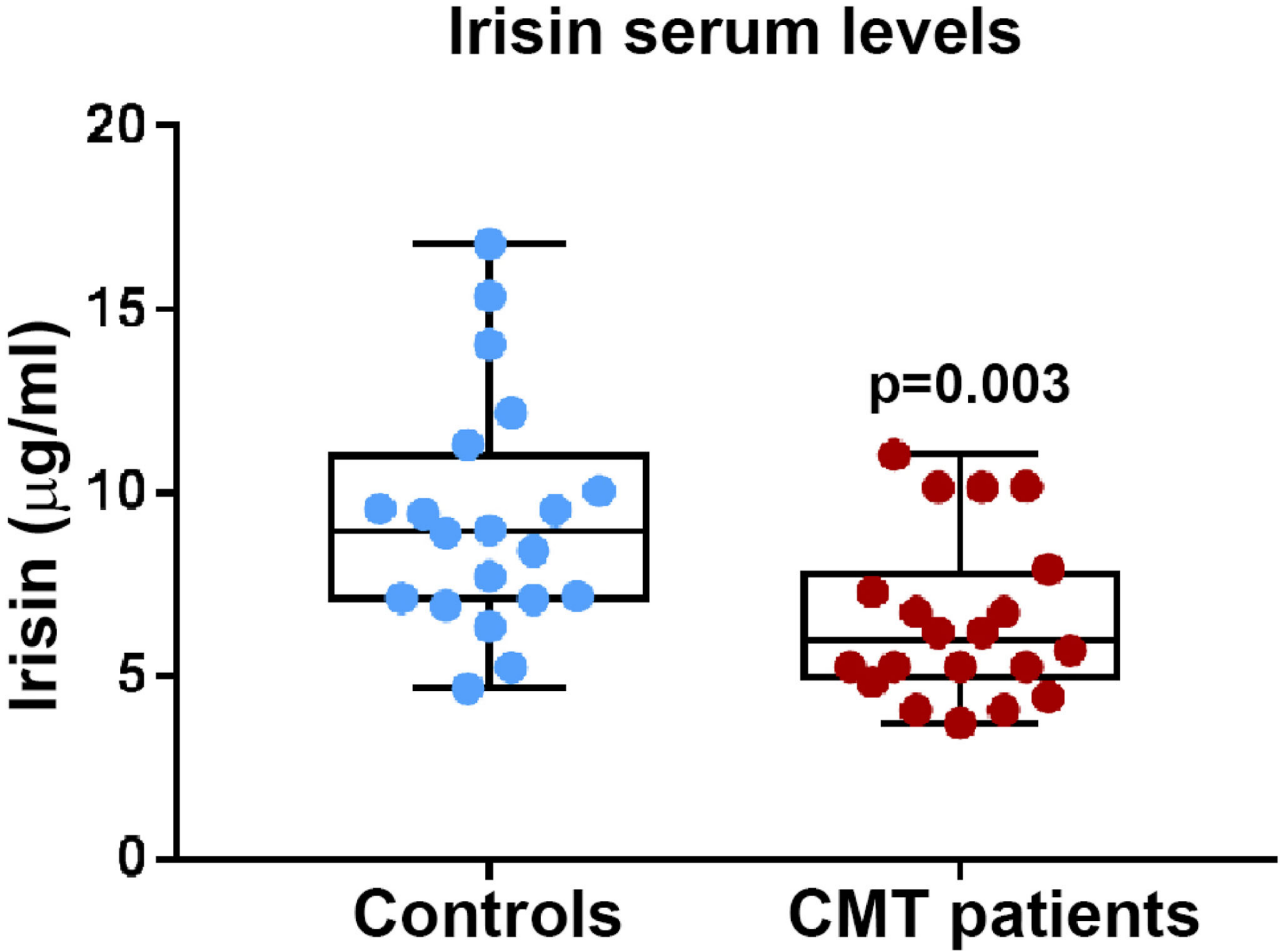
Irisin serum levels are significantly lower in CMT patients (N = 20) compared to healthy matched controls (N = 20); p values as indicated.

**Figure 2:**
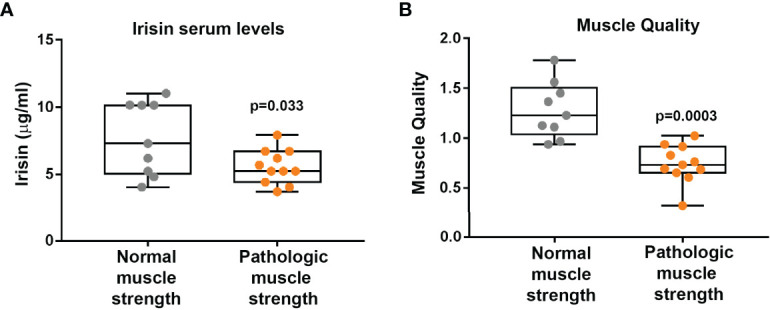
CMT patients with pathological muscle strength (N = 11) show lower Irisin levels than CMT patients with normal muscle strength (N = 9) **(A)**. Muscle quality is lower in CMT patients with pathological muscle strength (N = 11) than CMT patients with normal muscle strength (N = 9) **(B)**. Data are presented as box-and-whisker plots with median and interquartile ranges, from max to min, with all data points shown.

Before starting the evaluation of a possible influence of irisin on muscle strength and muscle mass, we compared irisin levels in CMT patients and control subjects matched for age and sex (N=20). Our findings demonstrate that CMT patients showed significantly reduced levels of circulating irisin compared to controls (6.51 ± 2.26 vs 9.34 ± 3.23 μg/ml; p=0.003) (**Figure 1**). In order to establish if irisin influenced muscle strength, we compared irisin serum levels in CMT patients sub-grouped in patients with normal (N=9) or low muscle strength (N=11) (**Figure 2A**). As shown in the **Figure 2**, serum irisin levels were significantly lower in the subgroup with low muscle strength than those of patients with normal muscle strength (5.56 ± 1.26 vs 7.67 ± 2.71 μg/ml; p=0.033). Interestingly, patients with low muscle strength showed also reduced muscle quality compared with those with normal muscle strength (0.74 ± 0.19 vs 1,28 ± 0,28; p=0.0003) (**Figure 2B**). As reported in the **Table 3**, a multivariate regression model in all CMT patients showed that irisin was, together with sex, the predictor of muscle quality (β=0.158; p=0.005) among the independent variables included in the model, such as 25(OH)-Vitamin D, myostatin and osteocalcin, which are well known to impact skeletal muscle function (26–28).

**Table 3:** Multivariate linear regression model to investigate the determinants of muscle quality; β and p values as indicated.

Dependent variable: Muscle Quality	β coefficient	p value
Age	-0.007	*0.32*
**Sex**	**-1.002**	***0.02***
SMM	-0.01	*0.54*
Vitamin D	-0.009	*0.27*
**Irisin**	**0.158**	***0.005***
Osteocalcin	0.00552	*0.67*
Myostatin	-0.0102	*0.60*

The bold values are statistically significant.

### Irisin Is Negatively Associated With Vitamin D Status in CMT Patients

**Figure 3:**
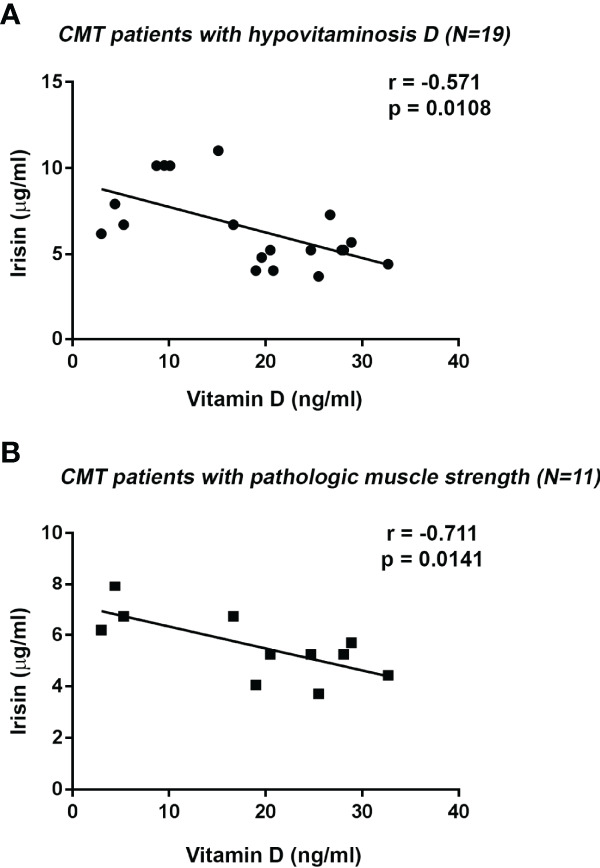
Negative correlation between Irisin and 25(OH)-Vitamin D serum levels in CMT patients with Vitamin D deficiency (N = 19) **(A)**. Irisin serum levels negatively correlated with 25(OH)-Vitamin D in CMT patients with pathologic handgrip (N = 11) **(B)**. Linear regression, r and p values as indicated.

As already described in **Table 2**, all CMT patients showed a negative correlation between serum levels of irisin and 25(OH)-Vitamin D (r=-0.526; 95%CI -0,791 to -0,095; p=0.017). This correlation was confirmed also after excluding the CMT patient not affected by vitamin D deficiency (r=-0.571; 95%CI -0.819 to -0.143; p=0.0108) (**Figure 3A**). Notably, we found a stronger inverse correlation between irisin and 25(OH)-Vitamin D by analyzing the subgroup of CMT patients with pathological muscle strength (r=-0.711; 95% CI -0,919 to -0,194; p=0.0141) (**Figure 3B**). In contrast, in the subgroup with normal muscle strength a correlation between irisin and 25(OH)-Vitamin D was found, but it did not reach statistical significance (r=-0.602; p=0.09).

### Irisin Positively Correlates With the Bone Formation Marker P1PN

**Figure 4:**
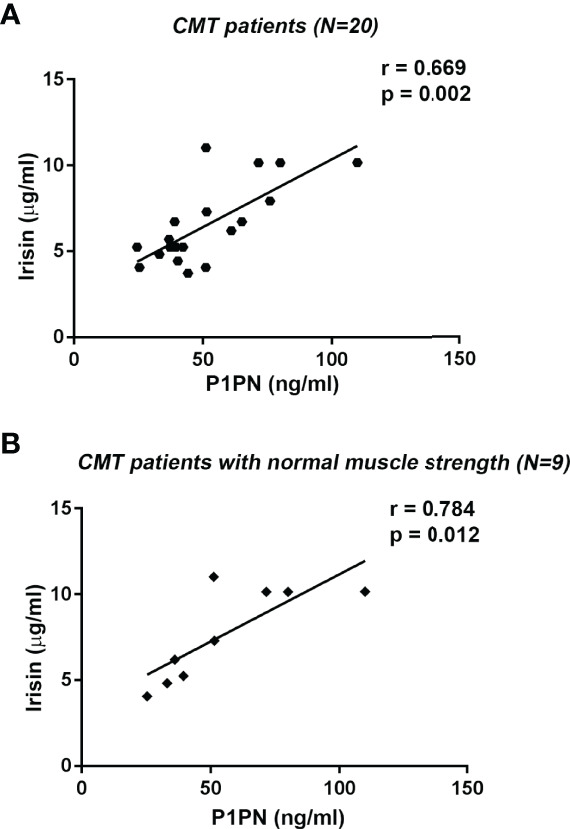
Linear regression (r and p values as indicated) showing positive correlation between Irisin and the bone formation marker P1PN in all CMT patients (N=20) **(A)** and in CMT patients with normal muscle strength (N=9) **(B)**.

Procollagen type I N-terminal propeptide (P1NP), the peptide cleaved from procollagen type I to produce type I collagen, is a widely used bone formation marker in clinical practice (29). In this cohort of CMT patients, P1PN values (median [IQR]: 43.27 ng/ml [37.06; 63.08]) were within reference levels (16-96 ng/ml). Analysis of all CMT patients showed a positive correlation between serum levels of irisin and P1PN (r= 0.669; 95% CI 0.295 to 0.865; p=0.002) (**Figure 4A**). Interestingly, we found a stronger positive association between irisin and P1PN by analyzing the subgroup of CMT patients with normal muscle strength (r= 0.784; 95% CI 0.251 to 0.952; p=0.012) (**Figure 4B**). In contrast, in the subgroup with pathologic muscle strength no correlation was observed. By performing a multivariate regression model in all patients, irisin was the only predictor of P1PN (β=5.257; p=0.011) among the independent variables included in the model (**Table 4**).

**Table 4:** Multivariate linear regression model to investigate the determinants of P1PN; β and p values as indicated.

Dependent variable: P1PN (ng/ml)	β coefficient	p value
Agee	-0.0827	*0.783*
Sex	-7.462	*0.481*
**Irisin**	**5.257**	***0.011***
Vitamin D	-0.261	*0.477*
Myostatin	-0.278	*0.746*

The bold values are statistically significant.

### Different Level of Irisin Between Male and Female Patients

**Figure 5:**
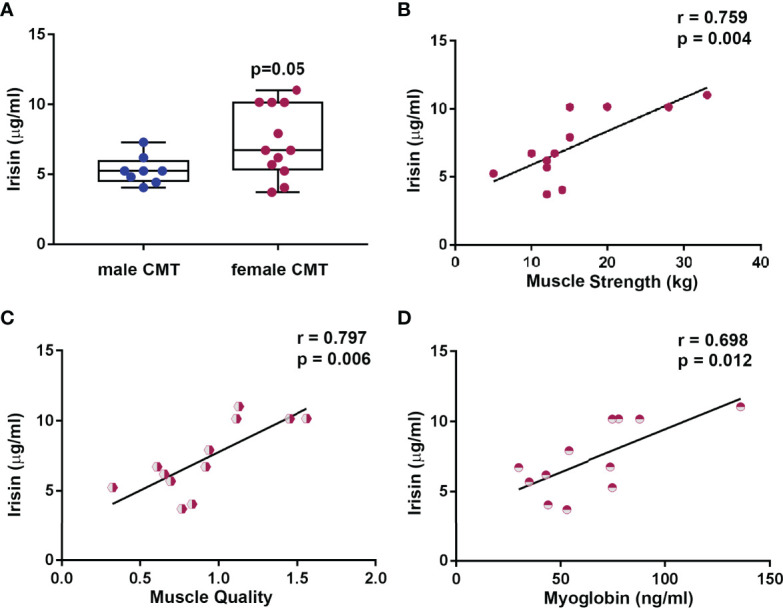
Circulating Irisin levels are higher in female CMT patients (N = 12) than in male (N = 8) **(A)**. Data are presented as box-and-whisker plots with median and interquartile ranges, from max to min, with all data points shown. In female CMT patients, Irisin positively correlated with muscle strength **(B)**, muscle quality **(C)**, and myoglobin **(D)**. Linear regression, r and p values as indicated.

Circulating Irisin levels were slightly higher in women than in men (7.31 ± 2.53 vs 5.31 ± 1.02 μg/ml, p=0.05) (**Figure 5A**). Considering data showing a robust association between irisin and muscle characteristics, we sought to determine if there was a different impact depending on gender. The analysis showed that only in the women irisin positively correlated with muscle strength (r=0.759; 95%CI 0.329 to 0.929; p=0.004) (**Figure 5B**), muscle quality (r=0.797; 95%CI 0.337 to 0.950; p=0.006) (**Figure 5C**), and myoglobin (r=0.698; 95%CI 0.207 to 0.908; p=0.012) (**Figure 5D**).

## Discussion

Currently, there is compelling evidence for a link between the myokine irisin and its positive effects on skeletal muscle mass. To the best of our knowledge, there have been no studies so far investigating circulating irisin levels in patients suffering from the CMT disease, whose clinical symptoms are often characterized by muscle atrophy (30). Our results reveal that irisin levels in patients with CMT are significantly lower than healthy controls matched for age, sex and BMI. There was a high prevalence (95%) of below-normal muscle quality, which, being expression of muscle strength/muscle mass, better describes the functional condition of the muscle compared to the two parameters taken individually. In order to assess the impact of irisin on skeletal muscle characteristics, we subcategorized patients based on their muscle strength evaluation. Interestingly, patients with pathological muscle strength showed reduced levels of circulating irisin compared to CMT patients with normal muscle strength, suggesting that irisin might represent a biomarker of muscle function. Interestingly, our regression analysis model showed irisin was the only predictor of muscle quality compared with all other independent variables, indicating that its circulating levels would help in monitoring the loss of muscle mass and strength in CMT patients.

Surprisingly, we found no correlation between irisin and myostatin levels in this study, although previous studies in animal models have demonstrated the existence of an interplay between these two myokines (31, 32). However, few data in humans have confirmed these mechanisms. Rather, a recent prospective and controlled clinical trial, aiming to evaluate the effects of exercise on circulating irisin and myostatin in older adult subjects, found no significant correlation between these myokines (33). Moreover, the study demonstrated that circulating irisin, but not myostatin, was a marker for improved muscle strength after resistance exercise (33). For a long time, the discovery of myostatin as a potent negative regulator of muscle mass has generated expectation that myostatin inhibition might be a therapeutic option for improving muscle mass in a broad spectrum of conditions characterized by muscle damage. Recently, the drug ACE-083 (34), designed to act locally *via* intramuscular administration, effectively induced localized muscle hypertrophy and improvement in force generation without systemic effects in wild-type mice and mouse models for CMT (34). Unfortunately, phase 2 clinical trials in patients with CMT have recently been discontinued due to failure in achieving significant improvements in muscle function, thus suspending further development of ACE-083 (35). Considering these results, it is not surprising that our multiple linear regression model showed that irisin, but not myostatin, was the independent variable that predicts muscle quality.

Another interesting finding from our study is that 95% of the patients included in our cohort had Vitamin D deficiency. Currently there are no studies reporting this incidence in relation to CMT disease. However, hypotheses have been postulated according to which vitamin D may have an impact on disease progression (36). In agreement with this hypothesis, there is a recent case report analyzing three cases of CMT patients, belonging to the same family, which shows that all patients had 25(OH)-vitamin D levels below or at the minimum threshold of the normal range (37). We previously observed a negative association between irisin and 25(OH)-Vitamin D in children and adolescents with type 1 diabetes mellitus (13). More recently, we found that pediatric patients with Prader Willi syndrome, not supplemented with 25(OH)-Vitamin D, showed lower irisin levels than both controls and supplemented patients (38). However, further studies are needed to understand the relationship between these two molecules and whether this interaction is influenced by disease type.

Consistent with the results of our study, it is plausible that low irisin levels, as well as Vitamin D levels, may also influence the bone mass of these CMT patients. Probably, CMT disease, being an inherited disease, whose onset occurs during childhood, interferes with the acquisition of the final peak bone mass. However, even in the absence of early onset, patients with CMT have impaired locomotion, leading to disuse osteoporosis (16). In this regard, a retrospective cohort study showed that patients with CMT had a 1.5-fold increased risk of fractures, primarily in the hands, feet, and ankles (39). By high-resolution peripheral computed tomography, it has been also demonstrated that three family cases of CMT showed severe deterioration of the trabecular compartment in the tibia (37). Of note, we observed a positive association between irisin and the bone formation marker P1PN in all patients, implying that higher circulating levels of myokine might positively impact bone mass. Consistently, our multiple linear regression model shows that irisin is a positive determinant of P1PN. In favor of this hypothesis, numerous evidences showed that irisin influences bone mass in children (12, 13), young adults (11, 40), and the elderly (15).

A limitation of our study is certainly the lack of bone mineral density measurement by dual-energy X-ray absorptiometry (DXA). Even though P1PN and CTX levels, biomarkers of bone formation and resorption respectively, were in the normal range, these data were not sufficient to draw conclusions about bone remodeling in the absence of the gold standard diagnosis by DXA. Furthermore, the effect of menopause, gender and age on bone metabolism markers must be considered. In particular, the age range of our cohort is very wide (20-76 years), also including menopausal women. Indeed, the levels of these markers are higher in men than in women in young adults, and decrease reaching their lowest levels in the fourth decade in women and in the fifth decade in men. Thereafter, both serum P1NP and CTX-I increase at menopause, while they remain stable or only slightly increase in men after age 70 (29).

Another weakness of our study relates to the absence of genetic test in some of the patients to establish the CMT subtype, and thus assessing the severity of the disease and how much it impacts on their mobility, since Irisin is released following muscle contraction, hence it depends on physical performance. Finally, we found a slight difference in irisin between men and women, however, limited by the small number of subjects included in our study, we cannot draw conclusions about a possible gender-specific effect of irisin on muscle quality in CMT, so further studies would be needed.

Overall, our results indicate that Irisin correlates with muscle quality and the bone formation marker P1PN in CMT patients and it could represent a new biomarker of the loss of muscle mass and strength. Further investigation is encouraged to confirm the promising potential of irisin as a new therapy for improving muscle strength and musculoskeletal function in CMT patients. Nevertheless, it is desired that scientific research will proceed to understand the role of irisin in the central and peripheral nervous system. Irisin has been detected in several brain regions (41), but also in the peripheral myelin sheath of muscle nerves (42). Numerous studies have focused on the efficacy of irisin in delaying the onset of neurodegenerative diseases, showing that increased irisin secretion promotes the release of brain-derived neurotrophic factor (43) and reduces β-amyloid deposition in the hippocampus (44). Further studies may uncover a possible involvement of irisin in preventing motor nerve fiber degeneration that occurs in CMT disease.

## Data Availability Statement

The raw data supporting the conclusions of this article will be made available by the authors, without undue reservation.

## Ethics Statement

The studies involving human participants were reviewed and approved by Ethics Committee of Bari University Hospital (Project Iri-mCMT N. 6012) Registered on www.ClinicalTrial.com (NCT04786522). The patients/participants provided their written informed consent to participate in this study.

## Author Contributions

Study design: GCo, AO, MB, and MG. Data collection: MD, RL, AF, FS, and AI. Data analysis: GCo, AO, GS, PP, and AI. Data interpretation: GCo, AO, MD, GCa, AL, MB, and MG. Drafting manuscript: GCo, AO, MB, and MG. Approving final version of manuscript: all authors.

## Funding

This work was supported by the Charcot Marie Tooth Italian Association (AICMT) and the grant “Tecnopolo per la Medicina di Precisione” D.G.R. n. 2117 of 21.11.2018 CUP B84I18000540002 to MG.

## Conflict of Interest

The authors declare that the research was conducted in the absence of any commercial or financial relationships that could be construed as a potential conflict of interest.

## Publisher’s Note

All claims expressed in this article are solely those of the authors and do not necessarily represent those of their affiliated organizations, or those of the publisher, the editors and the reviewers. Any product that may be evaluated in this article, or claim that may be made by its manufacturer, is not guaranteed or endorsed by the publisher.
