# Supplementary material for: Irisin Serum Levels and Skeletal Muscle Assessment in a Cohort of Charcot-Marie-Tooth Patients
Source: Front Endocrinol (Lausanne). 2022 May 12;13:886243. doi: 10.3389/fendo.2022.886243 (PMC9134857; doi:10.3389/fendo.2022.886243)
Supplement: Supplementary file 1 [file Table_1.pdf]

Cod.	CMT Type	Sex	Age (years)	Weight (Kg)	Height (cm)	BMI (kg/m^2)	SMM (Kg)	Muscle strength (Kg)	Muscle quality	bALP (µg/L)	TSH (µIU/ml)	Ldh (U/L)	Cpk (U/L)	Ca (mg/dl)	P (mg/dl)	Creatinine (mg/dL)	Myoglobin (ng/ml)	Haptoglobin (mg/ml)	25(OH)-Vit D (ng/ml)	CTX (ng/ml)	P1PN (ng/ml)	OPG (pmol/l)	RANK-L (ng/ml)	Osteocalcin (ng/ml)	Myostatin (ng/ml)	Sclerostin (pmol/L)
CMT-01	1A	M	75	71.80	158	28.76	24.40	30.00	1.23	21.40	1.90	178	180	8.70	2.90	1.02	114	6.71	27.90	0.072	39.45	2.90	12.55	10.90	4.89	129.26
CMT-02	N/A	F	20	48.50	156	19.93	14.20	13.00	0.92	14.94	1.17	155	58	9.30	4.40	0.53	30	7.59	16.67	0.218	65.11	4.81	8.25	17.10	1.48	57.29
CMT-03	1A	M	67	84.80	170	29.34	29.30	40.00	1.37	18.60	0.85	150	152	9.10	3.20	0.96	100	8.16	19.60	0.182	33.11	6.21	8.97	22.40	5.66	88.66
CMT-04	1A	M	43	68.00	154	28.67	31.50	21.65	0.69	13.60	0.39	131	190	8.90	3.00	0.41	109	7.73	32.70	0.308	40.26	7.28	36.89	24.20	10.71	72.83
CMT-05	2F	F	44	125.60	158	50.31	29.30	33.00	1.13	13.20	1.95	177	306	8.80	3.70	0.63	136	8.73	15.10	0.183	51.24	5.84	6.98	10.30	5.24	48.64
CMT-06	2F	F	54	72.10	159	28.52	19.30	28.00	1.45	13.70	1.62	144	171	8.80	3.10	0.71	88	9.27	8.70	0.210	80.07	2.50	0.51	19.50	2.10	68.43
CMT-07	1A	F	55	65.70	163	24.73	12.80	20.00	1.56	30.40	2.41	191	104	9.20	3.50	0.63	78	2.62	9.50	0.259	109.99	3.50	18.85	29.80	10.15	64.04
CMT-08	1A	F	31	62.10	157	25.19	15.50	5.00	0.32	12.30	1.58	204	119	8.40	2.50	1.07	75	2.84	28.10	0.238	37.22	2.50	15.41	15.20	4.75	23.95
CMT-09	1A	F	50	84.20	153	35.97	18.40	12.00	0.65	21.80	1.34	190	82	9.30	4.00	0.72	43	8.21	3.00	0.212	61.05	3.62	6.48	20.60	3.34	41.46
CMT-10	N/A	M	65	83.00	172	28.06	24.60	18.00	0.73	12.30	1.19	171	145	9.00	3.30	0.59	105	5.38	24.70	0.279	42.33	5.81	33.84	19.40	3.30	72.48
CMT-11	N/A	M	41	87.10	178	27.49	29.20	52.00	1.78	19.90	1.40	155	206	9.30	3.40	0.98	66	8.28	26.70	0.284	51.48	4.99	23.99	22.20	3.22	68.75
CMT-12	1B	M	57	90.10	178	28.44	31.00	30.00	0.97	11.70	0.62	185	237	9.00	3.50	0.78	88	8.22	62.80	0.240	36.15	9.86	32.82	18.30	10.94	77.79
CMT-13	2F	M	70	81.70	162	31.13	25.40	26.00	1.02	10.40	0.99	150	273	8.90	3.20	0.67	147	7.96	20.50	0.089	24.47	6.60	36.97	10.70	19.83	111.52
CMT-14	1A	F	55	70.50	162	26.86	13.50	15.00	1.11	13.30	0.92	166	139	9.00	4.20	0.59	75	8.92	10.10	0.244	71.60	3.80	9.06	14.90	6.68	55.44
CMT-15	1A	F	53	64.50	165	23.69	17.30	12.00	0.69	27.50	0.63	159	62	8.90	3.30	0.60	35	7.01	28.90	0.305	36.91	7.30	1.41	21.20	5.05	95.22
CMT-16	1X	F	59	60.00	166	21.77	15.70	12.00	0.76	16.30	1.43	162	88	9.30	3.60	0.54	53	8.77	25.50	0.233	44.21	6.33	23.90	13.50	8.96	46.33
CMT-17	N/A	F	39	78.00	169	27.31	16.90	14.00	0.83	16.90	1.19	168	132	8.30	2.90	0.56	44	7.34	19.00	0.196	51.12	4.33	3.70	12.60	0.25	26.53
CMT-18	N/A	M	76	87.00	164	32.35	32.00	30.00	0.94	25.90	0.92	148	86	8.70	2.70	0.83	64	7.33	20.80	0.472	25.39	6.50	37.89	30.80	1.93	87.09
CMT-19	1B	F	59	79.00	164	29.37	16.50	10.00	0.61	21.10	2.44	99	107	8.70	3.90	0.63	74	8.70	5.30	0.397	39.02	4.53	2.25	27.50	6.94	79.11
CMT-20	1B	F	67	93.00	164	34.58	16.00	15.00	0.94	18.70	2.05	91	41	9.90	3.70	1.08	54	6.11	4.40	0.421	76.01	3.90	0.87	19.40	1.56	88.84

Supplementary Table 1. Demographic, anthropometric, skeletal muscle mass, and laboratory parameters described individually for each of the 20 patients.
